# An extended APOBEC3A mutation signature in cancer

**DOI:** 10.1038/s41467-021-21891-0

**Published:** 2021-03-11

**Authors:** Adam Langenbucher, Danae Bowen, Ramin Sakhtemani, Elodie Bournique, Jillian F. Wise, Lee Zou, Ashok S. Bhagwat, Rémi Buisson, Michael S. Lawrence

**Affiliations:** 1 Massachusetts General Hospital Cancer Center, Harvard Medical School, Boston, MA USA; 2 Department of Biological Chemistry, Center for Epigenetics and Metabolism, Chao Family Comprehensive Cancer Center, School of Medicine, University of California Irvine, Irvine, CA USA; 3 Department of Pathology, Massachusetts General Hospital, Harvard Medical School, Boston, MA USA; 4 Department of Chemistry, Wayne State University, Detroit, MI USA; 5 Broad Institute of Harvard and MIT, Cambridge, MA USA; 6 Department of Cancer Immunology, Institute for Cancer Research, University of Oslo, Oslo, Norway; 7 Department of Biochemistry, Microbiology and Immunology, Wayne State University School of Medicine, Detroit, MI USA; 8 Department of Pharmaceutical Sciences, School of Pharmacy and Pharmaceutical Sciences, University of California Irvine, Irvine, CA USA

**Keywords:** DNA, Cancer genomics

## Abstract

APOBEC mutagenesis, a major driver of cancer evolution, is known for targeting TpC sites in DNA. Recently, we showed that APOBEC3A (A3A) targets DNA hairpin loops. Here, we show that DNA secondary structure is in fact an orthogonal influence on A3A substrate optimality and, surprisingly, can override the TpC sequence preference. VpC (non-TpC) sites in optimal hairpins can outperform TpC sites as mutational hotspots. This expanded understanding of APOBEC mutagenesis illuminates the genomic Twin Paradox, a puzzling pattern of closely spaced mutation hotspots in cancer genomes, in which one is a canonical TpC site but the other is a VpC site, and double mutants are seen only in *trans*, suggesting a two-hit driver event. Our results clarify this paradox, revealing that both hotspots in these twins are optimal A3A substrates. Our findings reshape the notion of a mutation signature, highlighting the additive roles played by DNA sequence and DNA structure.

## Abstract (web summary)

The APOBEC mutation signature contributes to a significant percentage of human cancers. Here the authors via biochemical and computational analyses shed light on how DNA primary sequence and secondary structure jointly influence A3A substrate optimality.

## Introduction

Understanding the global and local genomic preferences of mutational processes enables elucidation of the biological history of tumors and helps to tell apart recurrent driver mutations that confer a fitness advantage from passenger mutations that are caused by predictable mutational mechanisms. Mutagenic processes in cancer leave DNA footprints called mutation signatures^1^, typically characterized by the type of basepair substitution (e.g., C:G to T:A), and the immediate flanking sequence context (e.g., trinucleotide). Mutation signatures have been crucially important in revealing a variety of endogenous and exogenous mutational processes in cancer, and have been useful in uncovering the biological origins and natural history of numerous tumor types. One of the most prominent mutation signatures in cancer, present in over half of human tumors, is called the APOBEC signature, or Signatures 2/13, and derives from the activity of the APOBEC3A (A3A) and APOBEC3B (A3B) cytosine deaminases, which preferentially deaminate cytosines in the context of an immediately preceding thymine (written TpC)^1,2^. A rush of recent reports has detailed the crucial importance of this signature in driving tumorigenesis and facilitating the emergence of drug resistance^3–6^. The APOBEC mutation signature was originally established based on the ability of A3A/A3B to deaminate cytosines in single-stranded unstructured DNA substrates^7,8^ or in short viral sequences^9^, and it has long been the established framework through which APOBEC3 mutagenesis is viewed. The characterization of APOBEC3 activity informed by unstructured templates has led to this family of enzymes being commonly referred to as the TC-specific APOBECs^10–13^, their deaminase activity defined as being TC-dependent^14^, and their mutational signature described simply as “the tell-tale 5′-TpC signature”^15^. This framework for understanding the APOBEC mutation signature(s) has proven useful in analyzing and explaining much of the endogenous APOBEC mutational landscape in cancer.

While the preference of A3A/A3B for the TC sequence motif is evident in tumors, structural features are also crucial to consider^16,17^. Hairpin structures assumed by RNA^18,19^, or by DNA^20,21^ while it is transiently single-stranded, e.g., during replication, can greatly increase their fitness as A3A substrates. Furthermore, the activity of A3A at these hairpin loci can be reliably predicted from features of the hairpin, namely stem strength, loop length, and the positioning of the TpC site within the loop. In particular, TpC sites at the 3′-end of triloops (3-nt loops) in very stably paired stem-loops were found to be hundreds of times more mutable than comparable linear substrates. This explained numerous recurrent mutation hotspots in cancer genomes (previously assumed to be driver events) as instead merely recurrent passenger events arising at perfect A3A substrates.

A particular class of twin mutation hotspots in APOBEC+ samples has eluded explanation, a puzzle that we refer to as the genomic Twin Paradox. In these twin pairs, one hotspot is clearly a perfect A3A substrate (suggesting it may be an A3A-induced passenger event), but the other hotspot is a non-TpC site (suggesting it is not an APOBEC substrate at all). Initial reports^22,23^ of these sites noted that they are separated by just 1 or 2 basepairs, are flanked by palindromic sequences, and are recurrently mutated in APOBEC+ tumors. An example occurs in the promoter of the gene *PLEKHS1*, where a pair of highly mutated hotspots are separated by just two basepairs. The fact that these mutations are located in a gene promoter lends weight to the idea that they might have a functional role. Indeed, recent work^24,25^ has focused on these twin hotspots mutations in *PLEKHS1* and *ADGRG6* (also called *GPR126*) as potential drivers or biomarkers of bladder cancer. In some cases, a pair of twin hotspots is so frequently mutated that we are able to observe some patients carrying mutations at both sites. These rare double-mutated tumors allow us an opportunity to observe whether the mutations occur on the same DNA allele (in *cis*), or on different alleles (in *trans*). Surprisingly, the mutations have been seen in *trans* in all observed cases^23^, an intriguing pattern that suggests a possible double-hit tumor suppressor mechanism, in which cancer cells achieve a fitness benefit from inactivating both copies of the gene. However, whether the twin hotspots are both drivers, or both passengers, or one of each, has remained a mystery.

Recognizing the important effect of DNA secondary structure on A3A substrate optimality, here we set out to revisit the traditional definition of the APOBEC mutation signature, this time agnostic of primary sequence, and without assuming that A3A acts on unstructured substrates. Our analyses reveal that VpC substrates (V = not T), when presented in an optimal hairpin context, can be excellent substrates for A3A, superior to many TpC substrates. Furthermore, we show that mutations at VpC sites in optimal DNA hairpins are highly enriched among mutations in A3A-positive cancers. We see this VpC hairpin signature as completing an extended APOBEC3A signature. Finally, we are able to resolve the genomic Twin Paradox, by revealing that actually both sites in the twin hotspots are structurally optimal A3A substrates, suggesting that these twin mutation hotspots are likely twin passenger events due to A3A activity.

## Results

### APOBEC3A is capable of deaminating VpC sites in DNA hairpins in vitro

**Fig. 1:**
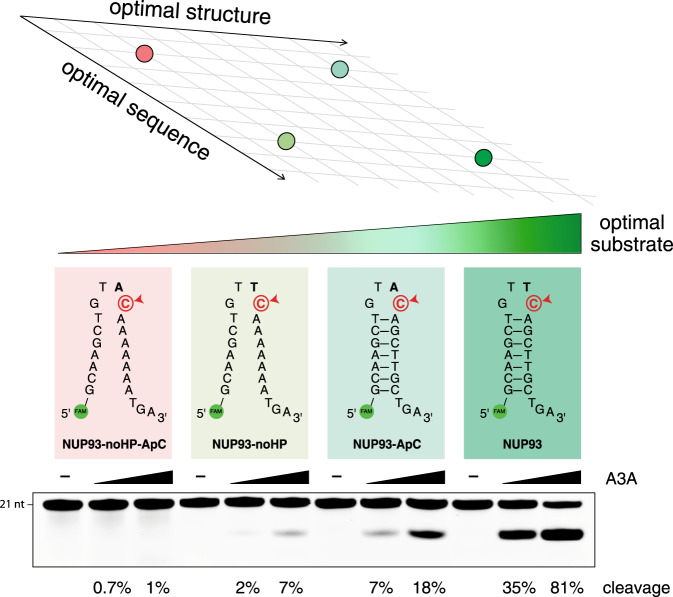
Primary sequence and secondary structure contribute additively to APOBEC3A substrate optimality. Four substrates are compared, each a version of the DNA hairpin site in the human *NUP93* gene. In vitro APOBEC3A activity assay shows that APOBEC3A substrate optimality increases as the DNA sequence improves in optimality (changing an ApC site to a TpC site), and also as the DNA structure improves in optimality (changing from a non-hairpin site to a hairpin site). Strongest activity is seen when both sequence and structure are optimal. Source data are provided as a Source Data file.

To systematically investigate the deamination activity of APOBEC3A on a variety of structured and unstructured substrates, we incubated extracts of APOBEC3A-expressing cells with a panel of DNA oligos and measured the catalytic activity of A3A in vitro^20^. Previous reports^1,2,10–15,26^ have established A3A’s strong preference for a 5′-T over all other preceding nucleotides when the target substrate is single-stranded. Our results confirm this: for example, an unstructured (non-hairpin) DNA substrate with a 5′-TC sequence showed moderate activity in the activity assay, but changing the 5′-TC to a 5′-AC abolished activity (Fig. 1). Furthermore, corroborating our previous work, A3A shows markedly stronger activity when the TpC is presented at the 3′-end of a short hairpin loop closed by a stably base-paired stem. This hairpin TpC site is a highly optimal substrate for A3A, as we previously reported^20^. However, we were surprised to see that when we tested a 5′-AC in the hairpin context, A3A showed significant and highly reproducible enzymatic activity, even higher than the activity on the non-hairpin 5′-TC (Fig. 1). We tested additional VpC hairpin sites and observed similar levels of activity (Supplementary Fig. 1A). These surprising results suggest orthogonal, compounding influences of having an optimal primary structure (local DNA sequence) and secondary structure (DNA hairpin) on A3A activity. Finally, to verify that A3A activity is truly the rate-limiting step in our in vitro assay and that the difference in A3A activity at DNA hairpins is not the result of differential activity of uracil DNA glycosylase (UDG) modulated by the structures of these substrates, we tested synthetic uracil-containing DNA substrates in the cleavage assay. Both a non-hairpin substrate and a hairpin substrate were cleaved quantitatively under the assay conditions (Supplementary Fig. 1B), confirming that our assay is not rate-limited by UDG activity, and provides a faithful readout of APOBEC activity levels.

### APOBEC+ tumors accumulate mutations at VpC sites in hairpin-forming sequences

**Fig. 2:**
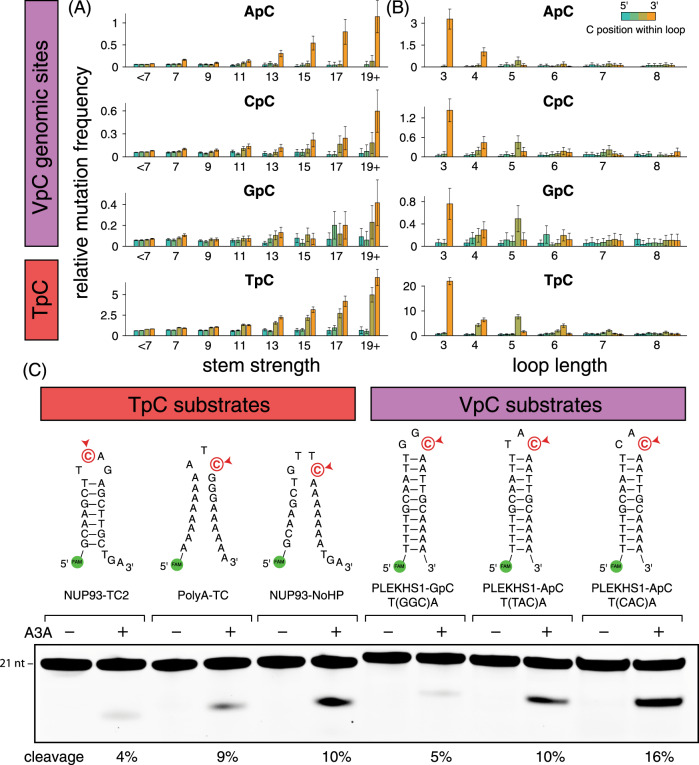
VpC sites and TpC sites show similar patterns of mutability. **A** Relative mutation frequency in APOBEC+ tumor samples increases with strength of hairpin base-pairing potential, both for TpC genomic sites, as well as for ApC, CpC, and GpC genomic sites (collectively known as VpC sites). Error bars represent 95% confidence intervals. **B** Mutation frequency decreases with size of hairpin loop, and requires optimal positioning of the cytosine within the loop, both for TpC and VpC sites. Error bars represent 95% confidence intervals. **C** In vitro APOBEC3A activity assay shows that APOBEC3A substrate optimality is jointly determined by sequence and structure, both for TpC and VpC sites. Source data are provided as a Source Data file.

The ability of A3A to deaminate non-TpC sites in DNA hairpins in vitro prompted us to reinvestigate whether C→ (T/G) mutations accumulate at non-TpC sites in hairpin-forming sequences in patients with high levels of APOBEC mutations. We analyzed a collection of published whole-genome and whole-exome-sequenced tumors from a variety of cancer types and assessed their mutational spectra and mutation rates in various genomic contexts. We concentrated on a set of patients with strong evidence of APOBEC-mediated mutational histories, using non-negative matrix factorization (NMF) to decompose mutation signatures^1,2^ and defining as APOBEC+ those tumors with at least 50% of their mutations assigned to the APOBEC mutation signature. We aggregated mutation statistics across disparate genomic sites sharing secondary structure characteristics regardless of the preceding nucleotide, in order to explore how hairpin-feature dependency of A3A mutability at TpC sites can be extended to VpC contexts as well. We showed in our previous work that APOBEC3A mutation frequency at TpC sites increases with hairpin stem strength (here defined as #AT basepairs + 3 × #GC basepairs). In our broadened analysis across all cytosines, we observed that this dependency holds true for VpC sites as well (Fig. 2A). We also observed the same preferences for smaller loop sizes and 3′-positioning within the loop for VpC sites as for TpC’s (Fig. 2B). Notably, all three possible VpC sequences (ApC, CpC, GpC) showed increasing mutation rate in strong hairpins (Supplementary Fig. 2A), and all 16 possible NNC triloops demonstrated the effect (Supplementary Fig. 2B). Overall, TpC sites have higher mutation rates than VpC sites in the same structural context. However, VpC sites in optimal hairpins displayed significantly higher mutation rates than TpC sites in linear DNA or suboptimal hairpins. These results confirm that the activity of A3A on a particular DNA substrate cannot be predicted simply by looking at the identity of the 5′ base, but instead both the primary and secondary structures must be considered.

To further investigate whether A3A could be responsible for VpC hairpin mutations in tumors, we tested a variety of additional TpC and VpC sites in our in vitro assay for A3A activity and showed that both classes of sites include examples of both good and poor substrates for A3A (Fig. 2C). Hairpins with a VpC sequence can be better substrates for A3A than linear substrates with a TpC sequence, or hairpins with a unfavorably positioned TpC sequence. Nevertheless, TpC sequences are always better substrates for A3A than VpC sequences in the same structural context.

Together, our computational analysis of the VpC mutations in APOBEC+ tumors and our in vitro biochemical studies suggest that a 5′-situated thymine is not absolutely necessary for making an A3A substrate. The strong structural preference of A3A for cytosines in optimal hairpins may override the sequence preference for TpC in vitro and in tumors.

Notably, many C→ T mutations in VpC hairpins were previously classified as due to the Aging signature^1^. Our results suggest that some of these mutations in APOBEC+ tumors arise from APOBEC activity. We computed the percent of C→ T mutations at VCG sequences in each tumor that were in highly optimal hairpins. This was usually low (<1%), but was elevated in a minority of tumors, reaching as high as 5% (Supplementary Fig. 3A). Of the 36 tumors above 1%, half were breast cancers, followed by five bladder tumors and three lung adenocarcinomas. These three tumor types are among the most highly APOBEC-enriched tumor types, supporting APOBEC mutagenesis as their source. Some of them have elevated expression of APOBEC3A and/or APOBEC3B, however some do not (Supplementary Fig. 3B). We showed recently^18^ that the APOBEC mutation signature is a poor predictor of APOBEC3A/B expression in tumors, because while APOBEC mutations are a permanent record in a cancer cell’s genome, APOBEC expression can be transient, returning to baseline after mutagenic episodes^4^.

Analyzing mutational strand asymmetry, we and others previously^27–30^ reported that APOBEC-associated TpC mutations are enriched on the replicative lagging-strand template (LGST), in comparison to the leading-strand template (LDST), suggesting that they occur during DNA replication. In contrast, there was little bias between the transcribed and non-transcribed DNA strands in transcribed regions of the genome. We repeated this analysis on APOBEC-associated VpC mutations (Supplementary Fig. 4) and found that they exhibited the same association with replication, but no associations with transcription or fragile sites^31^, the same pattern as APOBEC-associated TpC mutations, suggesting that both TpC and VpC mutations arise primarily during DNA replication.

### Mutations at VpC hairpin sites are due to APOBEC3A not APOBEC3B

**Fig. 3:**
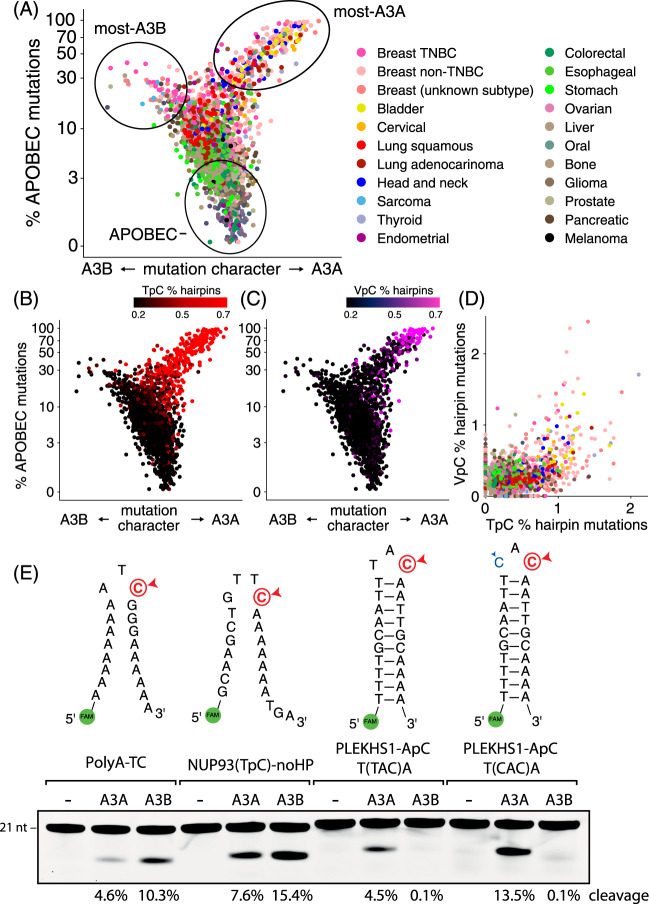
Hairpin mutations at VpC sites and TpC sites are caused by APOBEC3A not APOBEC3B. **A** A set of tumors sequenced by WGS was stratified by two parameters: the *y*-axis separates APOBEC− tumors (bottom) from APOBEC+ tumors (top), and the *x*-axis separates tumors with high A3B character (left) from those with high A3A character (right). Points are colored by tumor type (legend at right). **B** When tumors are colored by the fraction of APOBEC mutations occurring in TpC hairpins, it is clear that tumors enriched for TpC hairpin mutations (red) tend to be A3A-dominated APOBEC+ samples. **C** When tumors are colored by the fraction of APOBEC mutations occurring in VpC hairpins, the same pattern is evident: tumors with the highest levels of VpC hairpin mutations (magenta) tend to be A3A-dominated tumors. **D** TpC and VpC hairpin mutations are tightly associated: tumors enriched for one are enriched for both, suggesting that A3A is the joint cause of both. **E** Comparison of A3A and A3B activity on DNA oligos representing non-hairpin TpC sites and hairpin VpC sites. A3A activity is seen on all substrates, but A3B activity is not observed at VpC sites. Source data are provided as a Source Data file.

Having demonstrated the increased mutation rates of VpC hairpins in APOBEC+ tumors and the ability of A3A to modify VpC hairpins in vitro, we next asked whether A3A is the main driver of VpC mutations in hairpins in tumors. We previously^20^ characterized APOBEC+ tumors by their A3A vs. A3B character, using an established metric^32^. This yields a Y-shaped “bird plot” (Fig. 3A) that separates A3B-dominated tumors (left wing) from A3A-dominated tumors (right wing) from APOBEC-negative tumors (body). Mutations at TpC sites in hairpins are enriched in A3A-dominated tumors (red dots, Fig. 3B). Similarly, mutations at VpC sites in hairpins are also enriched in A3A-dominated tumors (magenta dots, Fig. 3C). Strikingly, no enrichment of mutations in hairpins is seen in A3B-dominated tumors, either for TpC hairpins (as previously reported^20^), or for VpC hairpins. Finally, we observed that tumors showing high enrichments of TpC hairpin mutations are exactly the same tumors showing high enrichments of VpC hairpin mutations (Fig. 3D), confirming that the two classes of hairpin mutations are tightly associated, and supporting A3A as the single cause of both.

Next, to confirm that A3A, and not A3B, generates mutations at VpC hairpin sites, we compared the in vitro activity of A3A and A3B on a panel of DNA oligonucleotides. We used non-hairpin TpC substrates to determine the amounts of A3A and A3B showing comparable levels of APOBEC activity (Fig. 3E, left two substrates). When these amounts of A3A and A3B were tested on hairpin VpC substrates, only A3A but not A3B showed activity (Fig. 3E, right two substrates). We also showed the same pattern using endogenous APOBEC activity. The human pharyngeal squamous carcinoma cell line BICR6 expresses low levels of A3A at baseline and showed low activity on a VpC hairpin substrate (Supplementary Fig. 5A, B). However, stimulating the cells with gemcitabine and interferon alpha (GEM/IFNα) dramatically increased A3A protein amounts and increased cleavage of VpC hairpin substrates by up to tenfold (Supplentary Fig. 5A–D). The effect was eliminated by co-administration of a small interfering RNA specifically against A3A (siA3A), returning cleavage levels to baseline. These results validate our finding that A3A is responsible for the observed activity at VpC hairpin sites. In contrast, the high levels of endogenous A3B in BICR6 cells showed no activity at VpC hairpin sites, but strong activity at non-hairpin TpC sites that was abrogated by treatment with an siRNA against A3B (Supplementary Fig. 5E–G). We confirmed this result using the human osteosarcoma cell line U2OS, which expresses no detectable A3A and high levels of A3B (Supplementary Fig. 5H). At a normalized level of A3B, cell extracts from U2OS and BICR6 cells showed similar activity at non-hairpin TpC sites, whereas U2OS cell extract showed no activity at VpC hairpin sites (Supplementary Fig. 5H, I).

### Human APOBEC3A displays activity at VpC sites in the *E. coli* genome

**Fig. 4:**
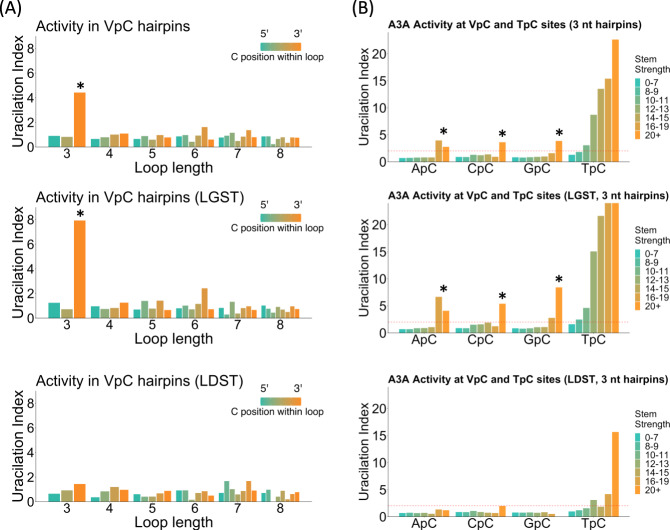
APOBEC3A can deaminate VpC sites in bacteria. **A** Relative APOBEC3A activity, measured as Uracilation Index (UI), at cytosines in VpC sequences in predicted hairpin loops in the genome of *E. coli*. Increased activity is observed at cytosines in NVC triloops (asterisks), but only on the lagging-strand template (LGST). **B** Comparison of relative APOBEC3A activity in VpC sequences. Increased activity is seen at VpC sites in the loops of strongly paired hairpins (asterisks). Activity at hairpin VpC sites is several times stronger than at non-hairpin TpC sites. Activity at hairpin TpC sites is much higher than comparable VpC sites. Source data are provided as a Source Data file.

Having observed A3A activity at non-canonical VpC sites in DNA hairpins in vitro and mutations at VpC sites in hairpin-forming sequences in tumors, we next investigated an in vivo laboratory model of A3A activity: bacterial cells expressing the human A3A enzyme. We described previously a method to enrich and sequence DNA fragments containing uracils (UPD-seq). We applied this method to A3A-expressing *E. coli* cells^21^. Uracils created by A3A at various genomic positions were quantified by computing the uracilation index (UI) which is a quantitative measure of the frequency at which any cytosine in the genome is converted to uracil by A3A. This analysis showed that the UI at TpC’s within predicted hairpin loops increased with stem strength and was highest for triloops with the target cytosine at the 3′ side of the loop (Supplementary Fig. 6A), exactly matching the patterns we reported previously for A3A activity in synthetic oligos and in human tumors. When these analyses were extended to VpC sequences in the *E. coli* genome, the highest UI values were found for VpC sites within hairpin loops with highest stem strengths and in which the cytosine was situated at the 3′ side of a triloop. VpC sites in strong hairpins showed four-times as much uracilation compared to non-hairpin VpC sites (Fig. 4A) and this effect was seen for all four possible 5′ nucleotides (Fig. 4B). This effect was not seen when analyzing cells expressing a catalytically inactive A3A point mutant or containing the empty vector (Supplementary Fig. 6B). This shows that while the magnitude of the UI is much greater for TpC sites in 3-nt hairpin loops than VpC sites even for strong hairpins (Fig. 4A, B, upper panels), A3A prefers cytosines in both these groups of sites over sites in larger loops. As noted previously^21^, the hairpin effect at TpC’s was stronger for cytosines located on the LGST during replication than the LDST (Fig. 4B, lower panels). Expanding the strand asymmetry analysis to VpC sequences, we found that this was also true for ApC, CpC, and GpC sites, increasing to eightfold for cytosines at the 3′ side of triloops (Fig. 4A, B, lower panels). Together, these results confirm that A3A can show activity at sites previously considered not to be substrates for A3A activity, namely VpC sequences presented in hairpin loops. We see this VpC hairpin signature as completing an extended APOBEC3A signature and provide a new software tool ApoHP for quantifying hairpins and hairpin mutations.

### Driver and passenger mutations at VpC sites in APOBEC+ tumors

**Fig. 5:**
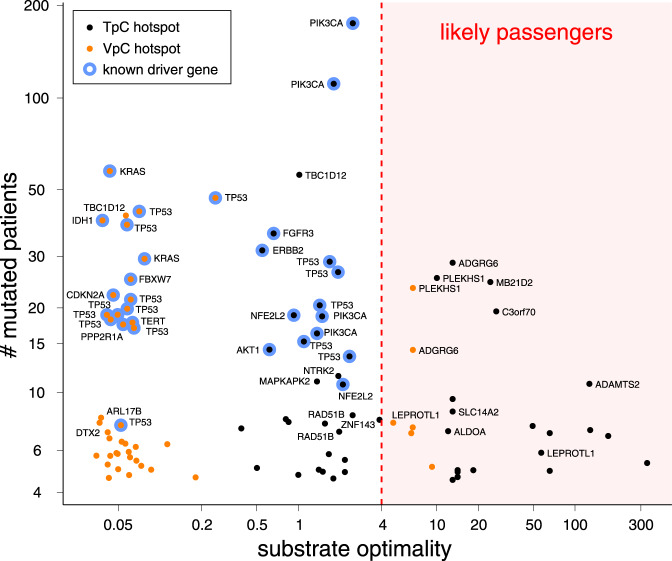
VpC passenger hotspots. The top 100 mutation hotspots in a set of APOBEC+ tumors. The *x*-axis represents substrate optimality, the expected mutation frequency relative to the baseline mutation rate at all linear TpC sites. The *y*-axis represents the number of patients in this cohort carrying a mutation at that site. Hotspots at optimal hairpin substrates for APOBEC3A (*x* ≥ 4) are likely passenger hotspots, whereas hotspots at ordinary sites (*x* < 4) are known or likely drivers. VpC hotspots in *PLEKHS1* and *ADGRG6* (also called *GPR126*) can now be understood to be optimal APOBEC3A hotspots despite their non-canonical primary sequence. Source data are provided as a Source Data file.

If VpC sites in DNA hairpins are indeed substrates for A3A in tumors, then one would expect to see recurrent mutations at these sites in APOBEC+ tumors. We surveyed the top 100 most frequently mutated C:G basepairs, including both TpC and VpC sites, in our APOBEC+ cohort (Fig. 5). As previously noted, many of the top recurrent TpC mutations are not in known driver genes, but are in optimal hairpins. These TpC sites are likely hotspots of passenger mutations due to their high substrate optimality for A3A. We also observe several VpC mutation hotspots in genes not known to confer a fitness advantage, at sites predicted to be highly optimal A3A substrates due to the favorable positioning of the cytosine at the 3′ end of a short loop closed by a strongly paired stem. For example, there are frequently mutated ApC hairpin sites in *ADGRG6* (also called *GPR126*) and *PLEKHS1*. These hotspots are likely favored targets of A3A, easy to mutate due to their optimal secondary structure. Additional examples of recurrently mutated VpC hairpins are shown in Supplementary Fig. 7. These hotspots illustrate that the substrate preferences of A3A in vivo are broader than initially thought, and that many of the most recurrently mutated loci in APOBEC+ tumors can be properly explained as passenger hotspot mutations driven by the preference of A3A for hairpins.

In contrast to the recurrent VpC mutations in optimal hairpins, many other recurrent VpC mutations are at non-hairpin sites in known driver genes like KRAS and TP53. These sites have low predicted substrate optimality relative to the background linear-substrate TpC mutation rate. They are ordinary sites without the special structural properties that make for an optimal A3A substrate.

### The extended APOBEC3A mutation signature helps explain the genomic Twin Paradox

**Fig. 6:**
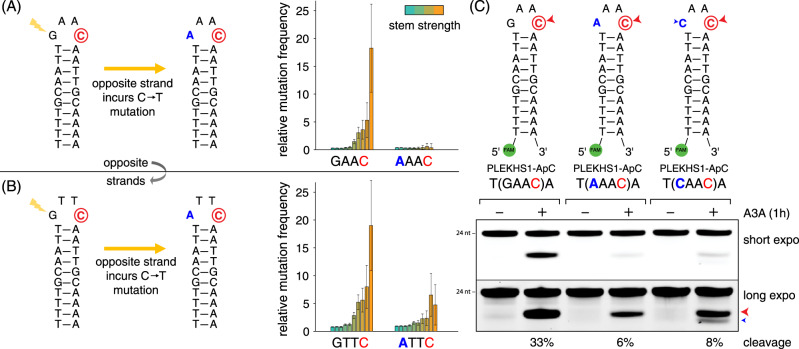
Twin mutation hotspot combining optimal TpC and VpC sites. A twin mutation hotspot detected in the gene *PLEKHS1* contains an optimal hairpin substrate on each strand of the DNA. **A** On one DNA strand, the hairpin contains the sequence GAAC, and the C in last position is an optimal VpC substrate for A3A. **B** On the other DNA strand, the hairpin contains the sequence GTTC, and the C in last position is an optimal TpC substrate for A3A. However, when A3A generates a mutation at either strand’s site, and the cell goes through DNA replication, then the mutation becomes propagated to the complementary strand, in the first (5’) position of the loop. This mutation causes the local sequence context to no longer be optimal, leading to a decrease in the observed mutation frequency in tumors (bar plots). Error bars represent 95% confidence intervals. **C** In vitro APOBEC3A assay confirms activity at GAAC hairpin loop sequence. Changing to AAAC or CAAC reduces the substrate optimality by corrupting the optimal GAAC sequence. Source data are provided as a Source Data file.

Our broadened definition of A3A substrate preferences sheds light on the genomic Twin Paradox, revealing that these twin hotspots are likely entirely APOBEC-caused. Specifically, our in vivo tumor statistics suggest a model in which the occurrence of the first mutation, followed by DNA replication and cell division, leads to propagation of the mutation to the opposite strand, resulting in a reduction of that strand’s fitness for A3A, and lowering the probability of incurring the second mutation (Fig. 6A, B). In short, the first mutation inhibits occurrence of the second. This model is supported by our in vitro biochemical experiments using a series of synthetic DNA oligos modeling the *PLEKHS1* twin hotspot (Fig. 6C). We find that the VpC site in the last position of the loop of the twin hotspot hairpins is most mutable with a guanine in the first position in the loop, i.e., in the reference configuration of the loop. Importantly, mutating the TpC nucleotide in the first position of the loop through A3A mutagenesis drastically reduces the mutability of the neighboring VpC site. Thus, these in vitro assays recapitulate the A3A-mediated mutagenesis at a twin hotspot, confirming that both the TpC and VpC sites are efficient substrates of A3A. Furthermore, when the mutation at the TpC is made permanent through DNA replication, it suppresses the occurrence of the mutation at the VpC site, explaining why these mutation pairs are seldom, perhaps never, observed in *cis* at the twin hotspot, and resolving the genomic Twin Paradox: these are simply pairs of closely situated A3A passenger hotspots.

## Discussion

Mutations caused by APOBEC enzymes are one of the most prevalent mutation signatures in cancer^26^ and have been studied intensively in recent years^3^. The signature has been known by its various separate aspects, such as its knack for generating strand-coordinated mutation clusters called *kataegis*^17,26,33,34^, its unusual tendency to generate mutations in gene-rich, early replicating, active chromatin^35^, its strand-asymmetric impact on the replicative LGST^27–30^, and its proclivity to mutate DNA and RNA hairpin loops^18–20,36^; but most of all, the APOBEC signature has been commonly understood as restricted to TpC sites^13–15^. Through our combined biochemical and bioinformatic analyses, we have shown that this understanding is incomplete: hairpin DNA sites can be optimal substrates for A3A even if they are not TpC sites. This reveals an extended APOBEC mutation signature that is broader than previously believed. In particular, many mutations previously thought due to other signatures, such as the Aging signature, may actually be due to APOBEC.

**Fig. 7:**
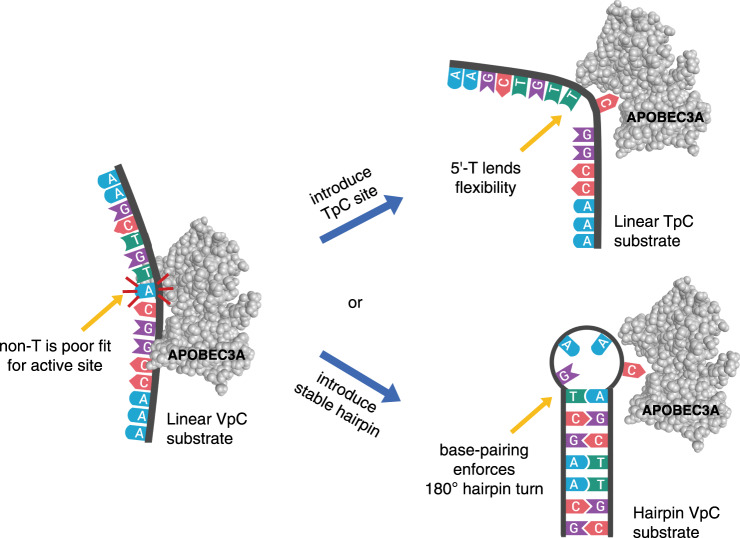
Determinants of APOBEC3A substrate optimality. Linear (non-hairpin) DNA substrate containing a VpC sequence has both non-optimal sequence and non-optimal structure, causing it to be a poor substrate for APOBEC3A. However, improving either the primary sequence (top path) or secondary structure (bottom path) improves the overall substrate optimality and enables APOBEC3A activity. Changing the VpC sequence to TpC enables productive contacts between the T and the enzyme, and may also increase flexibility at the precise point where the DNA needs to bend to fit into the A3A active site. Keeping the VpC sequence but changing the structure to a hairpin prepays the entropic cost of bending the DNA at the 5′ site, enforcing a 180-degree hairpin turn. (Figure created with BioRender.com).

The activity of A3A at cytosines other than TpC sites has escaped notice in most analyses of the APOBEC mutational signature because enzymatic activity at the vast majority of VpC sites is very weak, as most sites in the genome are not able to form a hairpin. However, when we focus our attention on hairpin sites, we are able to clearly see the activity of A3A at VpC sites. The preference for a T (and/or a hairpin turn) at the 5′-position may be due to the conformation of the target cytosine at the end of the loop. It has been shown that thymines contribute the least to base stacking^37,38^ and poly-dT is the most flexible homopolymer^39^. This flexibility may underlie A3A’s usual preference for a T preceding its substrate C. A3A may target linear TpC substrates due to the preceding T being able to move out of the way as A3A flips the neighboring cytosine into its active site. We suggest that the enforced sharp turn of a hairpin loop may prepay some of the entropic cost of this interaction (Fig. 7).

Our results in *E. coli* show that cytosines in hairpins are much more susceptible to deamination if they are on the LGST than the LDST, and this effect is strongest for cytosines at 3′ ends of triloops connected to stems with expected high stability. While the effect is seen with all C’s, it is much stronger for TpC’s than VpC’s. This is in agreement with our previous reports^27,28^ that APOBEC mutations are enriched on the LGST and this is true of mutations in both TpC and VpC sequence contexts (Supplementary Fig. 4). Our data suggest that at the replication fork, RPA (or SSB in *E. coli*) is unable to bind ssDNA that can form strong hairpins. RPA wraps a long stretch (~30 nt) of ssDNA around itself without using a high energy cofactor such as ATP^40,41^. Consequently, (1) high stem strength is essential to prevent binding of RPA: if a hairpin with high stem strength forms on the LGST, RPA would be unable to break the base pairing within the stem and bind the DNA; and (2) cytosines within loops of such hairpins are much more susceptible to deamination by A3A than those in random-coil ssDNA (Supplementary Fig. 8).

The greater susceptibility of cytosines in short loops to deamination by A3A probably reflects both (1) an intrinsic structural preference of A3A, as the sharp bending required to fit the substrate DNA into the active site of the enzyme^8,42^ is already present in short loops; as well as (2) a tendency of bases in short loops to flip out because of the strain and lack of base pairing. Previous work has posited that a preceding thymine may also play a role in stabilizing the aromatic residues in the hydrophobic binding pocket of A3A, implying that A3A has inherent sequence specificity in its binding capability^8^. However, cytosines in short loops (especially triloops) fit so well in the active site of A3A that the enzyme, even without hydrogen bonds to the 5′-T, can bind and deaminate them. This suggests that the catalytic advantage conferred by hairpin loops is an additive effect contributing to A3A substrate preferences, on top of underlying inherent primary sequence preferences. It is also possible that, as a member of an ancient family of zinc-dependent viral-defense enzymes, A3A evolved specifically for its ability to mutate hairpin loops, to aid in the recognition and defense against ssDNA viruses, which are known to adopt intricately folded structures^43^.

The observation of recurrent VpC mutations at poor A3A substrate sites in known driver genes in APOBEC+ tumors suggests that A3A may contribute to tumor fitness, although we cannot exclude the possibility that these mutations are generated by A3A-independent mechanisms. Recurrent VpC mutations at non-hairpin sites are found not only in known drivers, but also in genes not known to be drivers. These genes carrying recurrent VpC mutations at poor A3A substrate sites are possible novel drivers.

Understanding the factors that drive mutagenesis enables identification of driver mutations, which is crucial for the elucidation of the biological mechanisms of diseases and the efficient pursuit of novel therapeutics. Driver hotspots are specific genomic loci where mutations arise infrequently and stochastically, but are selected for and clonally expanded due to the strong fitness advantage they confer. In contrast, passenger hotspots^44^ are specific genomic loci where mutations occur frequently and predictably, but do not confer any fitness advantage. Distinguishing drivers from passengers is still not a fully solved problem in cancer genomics. We showed previously that A3A recurrently targets specific hairpin loci in the genome, leading to mutation hotspots in the absence of selective benefit. Our discovery that A3A can efficiently deaminate cytosines lacking a 5′-T if they occur in optimal hairpin structures allows us to explain the twin hotspot loci discussed above: these are a combination of two optimal substrates for A3A. These twin hotspots are fascinating bipartite mimics, pairs of sites that perfectly masquerade as two-hit tumor suppressors. Indeed, twin hotspot mutations in the gene *ADGRG6* (also called *GPR126*) were recently proposed^25^ as a potential novel driver of angiogenesis in bladder cancer. Our results show they are better understood as an exotic dually optimal mutagenic substrate. Other mutational processes are likely to recurrently target specific sequence/structure combinations, and understanding the causes of these passenger hotspots will help to separate true driver mutations from false-positive pseudo-drivers.

Separating true driver events from passenger mutation hotspots is crucial for effective analysis and clinical intervention in the diagnosis and treatment of cancer, particularly with the advent of targeted therapies. Knowing which mutations confer real fitness advantages to tumors and are thus worth investigating and targeting is an essential step when analyzing cancer genomic data. Furthermore, incorporating structural features into mutation signature analysis leads to a fundamentally expanded definition of an important endogenous mutational process. We anticipate that other forms of DNA structure, e.g., short cytosine-containing bulges or loops in structures like G-quadruplexes^45,46^ may also influence the activity of APOBEC and other mutational processes.

## Methods

### Plasmids

APOBEC3A and APOBEC3B cDNA were synthesized by GenScript with a beta-globin intron and a Flag tag at C-terminus. The plasmid expressing APOBEC3A-Flag, and APOBEC3B-Flag were generated by inserting the cDNA into pDEST53 vectors using the Gateway Cloning System (Thermo Fisher Scientific).

### Cell culture

HEK-293T cells were maintained in DMEM supplemented with 10% FBS and 1% penicillin/streptomycin. HEK-293T cells were transfected with pDEST53-APOBEC3A-Flag or pDEST53-APOBEC3B-Flag using Lipofectamine 2000 (Thermo Fisher Scientific). BICR6 was maintained in DMEM/F12 GlutaMAX^™^-I supplemented with 10% FBS and 1% penicillin/streptomycin. The cell lines above were purchased from either ATCC or Millipore-Sigma.

### Chemicals

Gemcitabine was purchased from Selleckchem and dissolved in DMSO. Purified human Interferon-αA/D was purchased from Millipore-Sigma.

### Cell extracts

HEK-293T cells or HEK-293T cells expressing A3A or A3B were lysed in 25 mM HEPES (pH 7.9), 10% glycerol, 150 mM NaCl, 0.5% Triton X-100, 1 mM EDTA, 1 mM MgCl2, and 1 mM ZnCl2 and protease inhibitors. Cell lysates were sonicated and centrifuged 10 min at 16,000 × *g* at 4 °C. Then, RNase A (0.2 μg/ml) was added for 30 min at 4 °C and cell lysates were centrifuged 10 min at 16,000 × *g* at 4 °C. Protein concentration of the supernatant was determined by Bradford assay (Bio-Rad).

### DNA deaminase activity assay

The deamination assays were performed as previously described^33,47,48^. Reactions (50 μl) containing 8 μl of a normalized amount of cell extracts (expressing A3A or A3B) were incubated at 37 °C during 1 h in a reaction buffer (42 μl) containing a DNA oligonucleotide (20 pmol of DNA oligonucleotide, 50 mM Tris (pH 7.4), 1.5 units of uracil DNA glycosylase (NEB), and 10 mM EDTA). Then, 100 nM of NaOH was added to the reaction following by 40 min incubation at 95 °C. Finally, 50 μl of formamide was added to the reaction (50% final) and the reaction was incubated at 95 °C for 10 min following by 5 min at 4 °C. DNA cleavage was monitored on a 20% denaturing acrylamide gel (8 M urea, 1X TAE buffer) and run at 65 °C for 80 min at 150 V. DNA cleavage of PLEKHS1- ApC-T(CAC)A oligonucleotide was monitored on a 24.5% denaturing acrylamide gel (8 M urea, 1X TAE buffer) and run at 65 °C for 4 h at 150 V. DNA oligonucleotide probes were synthetized by Thermo Fisher Scientific. The sequences of DNA oligonucleotide probes used in this study are listed in Supplementary Table 1.

### Bioinformatic analyses

The human genome (build hg19) was scanned for potential hairpin-forming sequences using the survey_hairpins function of the ApoHP tool [http://github.com/alangenb/ApoHP], which implements a version of the algorithm described in previous work^20^. Briefly, the genome was scanned for sequences of the form S-L-S′, where the sequences S and S′ are reverse-complementary with a sequence L (ranging from 3 to 11 nucleotides) intervening between them. Sequences such as these have the potential to form stem-loop, or “hairpin” structures in DNA that is transiently single-stranded. For each position p in the genome, flanking sequences S and S′ were sought such that position p would be in the intervening loop sequence L. Stem strength was defined as the number of A:T basepairs plus 3 × the number of G:C basepairs, an approximation of empirically measured nearest-neighbor stacking energies^49^. In cases where multiple alternative pairings were possible, the stem with the strongest pairing was chosen, using shortest loop size as a tie-breaker. The output of this procedure was to assign to each genomic position a set of parameters describing its hairpin characteristics: stem strength, loop length (in nucleotides), and position of the mutation-site cytosine within the loop (ranging from 1 to loop length). This allows genomic positions to be categorized into equivalence classes for investigating the influence of hairpin characteristics on relative mutation frequency, which we describe below.

Mutation calls from whole-genome sequencing (WGS) were obtained from TCGA and the International Cancer Genome Consortium Pan-Cancer Analysis of Whole Genomes project, and other projects^1,22^, as described below in the “Data availability” section. We restricted our analysis to somatic single-nucleotide variants (SSNVs) and excluded patients with fewer than 500 SSNVs in the genome, yielding a final WGS dataset comprising 2800 unique patients spanning 35 tumor types. Mutations were analyzed by the analyze_mutations function of ApoHP. Mutation signatures were analyzed by NMF, using *k* = 8, revealing a set of signatures corresponding to known mutational processes, including APOBEC enzyme activity. Each patient was characterized by *frac_apobec*, the fraction of its mutations assigned to the APOBEC signature. Cohorts of APOBEC+ patients were defined by imposing a threshold on *frac_apobec*. Our initial analyses used a set of 110 patients with *frac_apobec* ≥ 50%. We repeated some analyses using a cutoff of 10% or 90%, with similar results. To minimize the influence of potentially overlapping hypermutation processes such as MSI, Smoking, UV, POLE, or ESO, we excluded “MSUPE+” patients, those with at least 10% of their mutations assigned by NMF to one of these five other hypermutation processes.

To measure the joint influence of hairpin-forming potential and local sequence context on the relative mutation frequency of APOBEC signature mutations, we binned genomic cytosines into classes of equivalent cytosines that share hairpin characteristics and local sequence features. For each class of genomic cytosines, we counted the number of sites (*N*) in the genome that belong to this class, as well as the number of mutations (*n*) observed in the APOBEC+ cohort being analyzed. We then calculated the ratio (*n*/*N*), which represents the number of mutations per sites of this class. For classes of cytosines that have few sites in the genome and/or few observed mutations, the uncertainty on this estimated relative mutation frequency becomes large. Therefore, we estimated a 95% confidence interval on the ratio, by first approximating the standard deviation (SD) of the ratio by propagating an assumed square-root uncertainty in the integer counts, using the formula SD = (*n*/*N*) × [(*n*^½^/*n*)^2^ + (*N*^½^/*N*)^2^]^½^, and then estimating the 95% confidence interval as (*n*/*N*) ± 1.96 × SD. Finally, we normalized this ratio (and confidence interval) to a relative mutation frequency, by dividing the APOBEC+ cohort’s baseline rate of mutations at all TCA trinucleotides in the genome (the most favored site for APOBEC mutations). The bar plots in Figs. 2A, B and 6A, B and Supplementary Fig. 2A, B show this estimated relative mutation frequency, with the estimated 95% confidence interval shown as error bars. This analysis is part of the analyze_mutations function of ApoHP.

Quantitative modeling to predict *relrate_exp* for any given cytosine base in the genome was performed by the analyze_mutations function of ApoHP, and leveraged the observation that mutation rate in hairpin loops increases as an exponential function of stem strength^20^. We fit a global variable *m*_hp_ = the multiplicative increase in mutation frequency with each additional unit of stem strength. For each type of hairpin, defined by the loop length, loop position, and local sequence context, we fit a local parameter ss_o_ = the stem strength necessary to reach double the baseline non-hairpin mutation rate for that type of hairpin. For some hairpin types (e.g., with the C at the wrong side of the loop), mutation rate never rises above baseline, and ss_o_ is high. For optimal hairpins, mutation rate rises very quickly with increasing stem strength, and ss_o_ is low.

### Uracil pull-down and sequencing of bacterial genomic DNA

Wild-type A3A, its E72A mutant, or the empty vector were introduced in an *ung*^−^ *mug*^−^ strain of *E. coli*, and uracil-containing genomic DNA fragments were pulled-down and sequenced (UPD-seq) in previously published work^21^. Briefly, the genomic DNA was isolated and uracils were excised using *E. coli* Ung enzyme, and the resulting abasic sites were labeled using a chemical, ssARP, that contains a biotin. These labeled fragments were separated from the non-uracil-containing fragments using streptavidin beads, chemically cleaved off the beads and subjected to NextGen sequencing. The sequencing reads are available at the NCBI SRA (short read archive) under BioProject ID: PRJNA448166 (https://www.ncbi.nlm.nih.gov/bioproject/PRJNA448166/). The UI was defined as the fraction of the sequencing reads at a genomic reference cytosine that are called as thymine, multiplied by 1000. The potential hairpin loops in the *E. coli* genome were predicted using the same criteria used for the cancer genomes, and UI was calculated for predicted hairpin loops with different stem strengths, loop sizes, and loop sequences^21^.

### Statistics and reproducibility

All western blots and DNA gels were repeated at least three times and representative images are shown in this paper.

## Supplementary information

Supplementary Information

## Data Availability

This study analyzed published data and did not generate any new sequencing data. Sequencing reads from the uracil pull-down experiments^21^ are available at the NCBI SRA (short read archive) under BioProject ID: PRJNA448166. Mutation calls from TCGA whole-exome sequencing (WXS) were obtained from the TCGA Unified Ensemble MC3 Call Set^50^, the public, open-access dataset of somatic mutation calls produced by the MC3 calling effort (Multi-Center Mutation Calling in Multiple Cancers), downloaded from the following link: [http://www.synapse.org/#!Synapse:syn7214402/wiki/405297] (The results here are in whole or part based upon data generated by the TCGA Research Network: [http://cancergenome.nih.gov/] as outlined in the TCGA publications guidelines [http://cancergenome.nih.gov/publications/publicationguidelines]). Following the filtering procedure that was used for the PanCanAtlas project, the MC3 dataset was filtered to include only PASS variants, which removes patients that were subjected to whole-genome amplification (WGA), as well as the acute myeloid leukemia (LAML) cohort. This yielded a final cohort of 9023 patients covering 32 tumor types. Mutation calls from whole-genome sequencing (WGS) from TCGA and other projects were obtained from the International Cancer Genome Consortium (ICGC) Pan-Cancer Analysis of Whole Genomes (PCAWG) project. Mutation calls were downloaded from the ICGC Portal (https://dcc.icgc.org/api/v1/download?fn=/PCAWG/consensus_snv_indel/final_consensus_passonly.snv_mnv_indel.icgc.public.maf.gz and https://dcc.icgc.org/api/v1/download?fn=/PCAWG/consensus_snv_indel/final_consensus_snv_indel_passonly_icgc.public.tgz). Note that controlled tier access credentials are required from the ICGC and TCGA projects as described on the ICGC PCAWG site [http://docs.icgc.org/pcawg/data/]. Additional WGS data were obtained from published projects^1,22^ from the following links: [ftp://ftp.sanger.ac.uk/pub/cancer/AlexandrovEtAl/somatic_mutation_data] and [ftp://ftp.sanger.ac.uk/pub/cancer/Nik-ZainalEtAl-560BreastGenomes/Caveman_560_20Nov14_clean.txt]. Identifying and removing duplicate patients, restricting to somatic single-nucleotide variants (SSNVs), and excluding patients with fewer than 500 SSNVs in the genome yielded a final WGS dataset comprising 2800 unique patients spanning 35 tumor types. Gene expression measurements from TCGA RNA-Seq were obtained from the Broad Institute GDAC website [http://gdac.broadinstitute.org/runs/stddata__2014_09_02/data]. Source data are provided with this paper.

## Source data

Source Data

## Code availability

Source code and executable software tool ApoHP are available at http://github.com/alangenb/ApoHP and have been archived via Zenodo^51^.
