# Supplementary material for: An extended APOBEC3A mutation signature in cancer
Source: Nat Commun. 2021 Mar 11;12:1602. doi: 10.1038/s41467-021-21891-0 (PMC7952602; doi:10.1038/s41467-021-21891-0)
Supplement: Supplementary file 1 — Supplementary Information [file 41467_2021_21891_MOESM1_ESM.pdf]

Supplementary Information for

An extended APOBEC3A mutation signature in cancer

Adam Langenbucher, Danae Bowen, Ramin Sakhtemani, Elodie Bournique, Jillian F. Wise,

Lee Zou, Ashok S. Bhagwat, Rémi Buisson, Michael S. Lawrence

Supplementary Table 1. The sequences of DNA oligonucleotide probes used in this study.

PolyA-TC: 5’-(6-FAM)-AAAAAAAAATCGGGAAAAAAA

NUP93: 5’-(6-FAM)-GCAAGCTGTTCAGCTTGCTGA

NUP93-ApC: 5’-(6-FAM)-GCAAGCTGTACAGCTTGCTGA

NUP93-noHP: 5’-(6-FAM)-GCAAGCTGTTCAAAAAAATGA

NUP93-noHP-ApC: 5’-(6-FAM)-GCAAGCTGTACAAAAAAATGA

NUP93-TC2: 5’-(6-FAM)-GCAAGCTTCAGAGCTTGCTGA

NUP93-G(AGTC)C: 5’-(6-FAM)-GCAAGCGAGTCCGCTTGCTGA

NUP93-G(GGTC)C: 5’-(6-FAM)-GCAAGCGGGTCCGCTTGCTGA

NUP93-T(AGTC)A: 5’-(6-FAM)-GCAAGCTAGTCAGCTTGCTGA

NUP93-T(GAAC)A: 5’-(6-FAM)-GCAAGCTGAACAGCTTGCTGA

NUP93-G(GAAC)C: 5’-(6-FAM)-GCAAGCGGAACCGCTTGCTGA

NUP93-C(TAGC)G: 5’-(6-FAM)-GCAAGCCTAGCGGCTTGCTGA

PLEKHS1-GpC-T(GGC)A: 5’-(6-FAM)-TTTTGCAATTGGCAATTGCAAAA

PLEKHS1-ApC-T(TAC)A: 5’-(6-FAM)-TTTTGCAATTTACAATTGCAAAA

PLEKHS1-ApC-T(CAC)A: 5’-(6-FAM)-TTTTGCAATTCACAATTGCAAAA

PLEKHS1-ApC-T(AAC)A: 5’-(6-FAM)-TTTTGCAATTAACAATTGCAAAA

PLEKHS1-ApC-T(GAAC)A: 5’-(6-FAM)-TTTTGCAATTGAACAATTGCAAAA

PLEKHS1-ApC-T(AAAC)A: 5’-(6-FAM)-TTTTGCAATTAAACAATTGCAAAA

PLEKHS1-ApC-T(CAAC)A: 5’-(6-FAM)-TTTTGCAATTCAACAATTGCAAAA

PLEKHS1-GTTU 5’-(6-FAM)-TTTTGCAATTGTTUAATTGCAAAA

ssDNA-GTTU 5’-(6-FAM)-TTACGCAATTGTTUAAACTAACGT

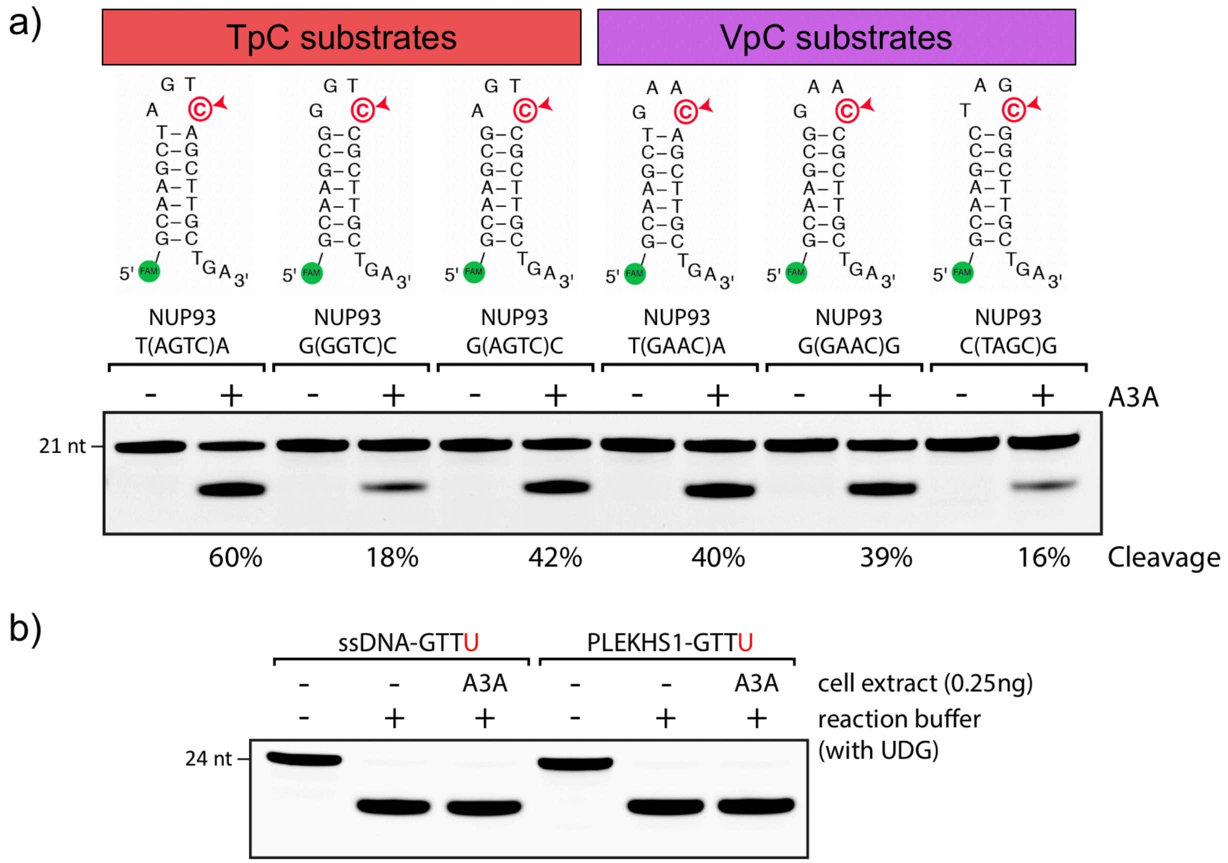

Supp. Fig. 1. DNA hairpins as optimal APOBEC3A substrates. (a) Both TpC and VpC hairpins can be optimal APOBEC3A substrates. A panel of six DNA oligos was tested *in vitro* as substrates for A3A. Three contained a TpC site in the hairpin loop, and the other three a VpC site. All six hairpins were efficient substrates for A3A, demonstrating that the identity of the 5' base (TpC or VpC) is not the only determinant of APOBEC activity. **(b)** Uracil excision by uracil DNA glycosylase (UDG) is not rate-limiting in our cleavage assay. Fully deaminated substrates were represented by synthetic DNA substrates with the target cytosine replaced by uracil (thus bypassing the APOBEC-dependent deamination step). Both a non-hairpin substrate (ssDNA-GTTU) and a hairpin substrate (PLEKHS1-GTTU) were 100% cleaved under the assay conditions, and the UDG in the reaction buffer was sufficient to support full cleavage; inclusion of cell extract was not required. Source data are provided as a Source Data file.

a)

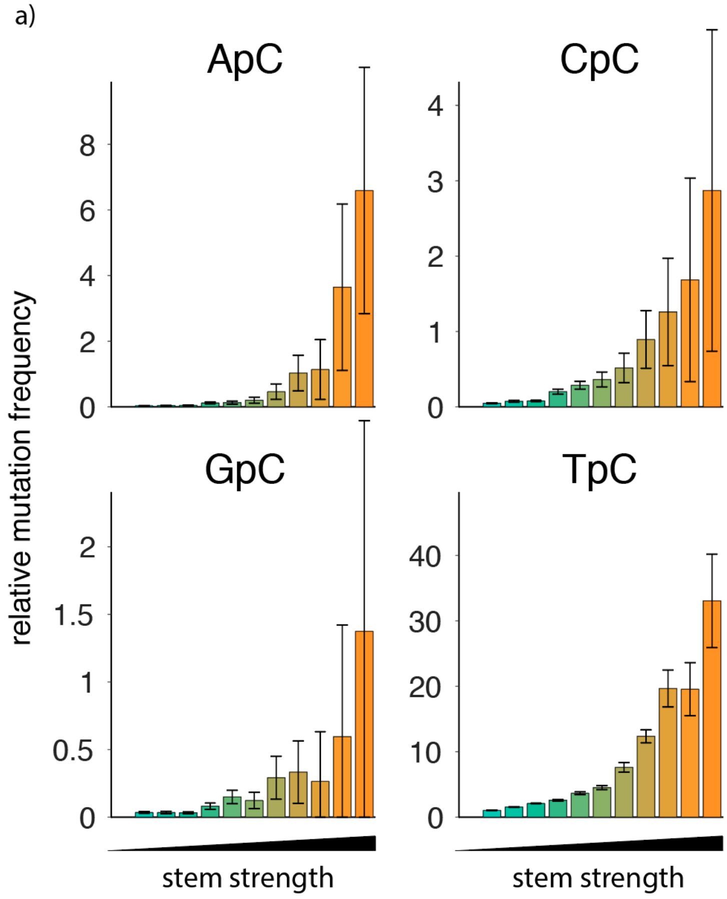

Supp. Fig. 2. APOBEC+ tumors show increased mutation frequency in hairpins, at both VpC and TpC sites. (a) Genomic cytosines were binned by the identity of the 5' base and the strength of potential base-pairing of the flanking sequences (hairpin strength). Relative mutation frequency was measured in each bin and normalized to the non-hairpin baseline for each category of sites. Error bars represent 95% confidence intervals. In all four cases (ApC, CpC, GpC, TpC), a systematic increase in mutation frequency was observed as hairpin strength increased. In this panel, our APOBEC+ cohort included all tumors with at least 10% of their mutations assigned by NMF to the APOBEC mutation signature. **(Supp. Fig. 2 continues on next page)**

b)

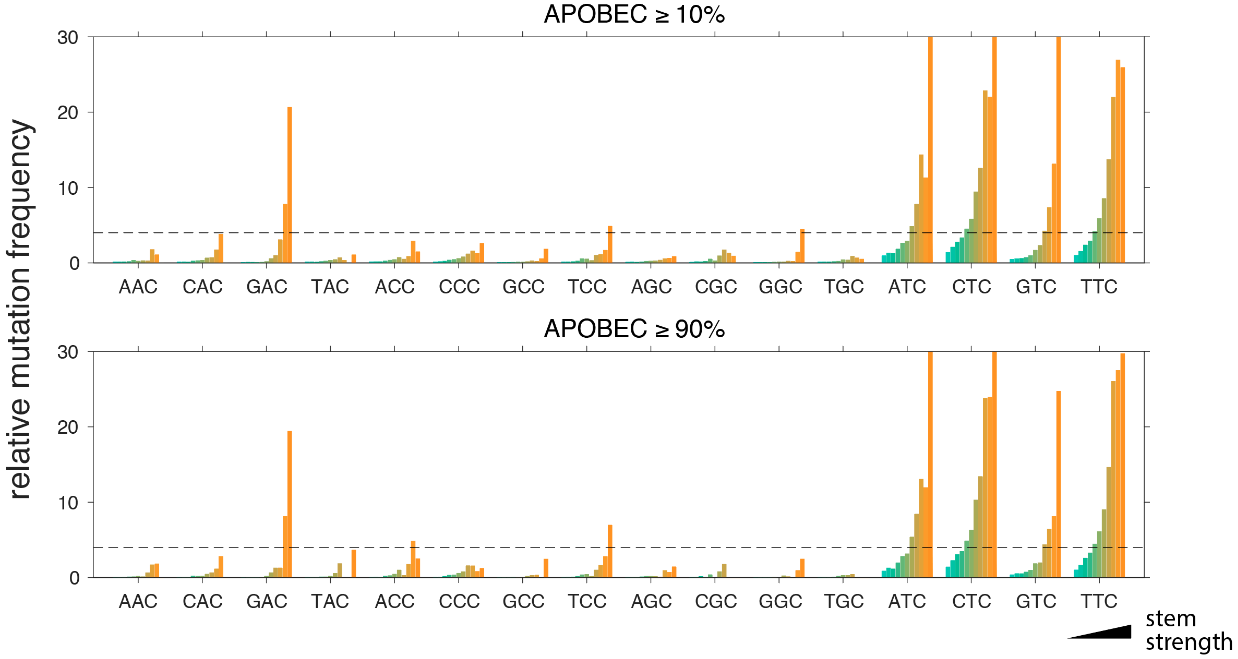

Supp. Fig. 2. (continued) **(b)** Genomic cytosines in 3-nt (NNC) hairpin loops were binned by the identity of the other nucleotides in the loop (16 possibilities) and hairpin strength, as in panel (a). Hairpin-dependent increases in mutation frequency were seen for each possible loop sequence. Two versions of the analysis showed concordant results: one including all patients with at least 10% of their mutations assigned to APOBEC (top row), and a more restricted analysis including only the purest-APOBEC patients, defined as those with at least 90% of their mutations assigned to APOBEC (bottom row). Source data are provided as a Source Data file.

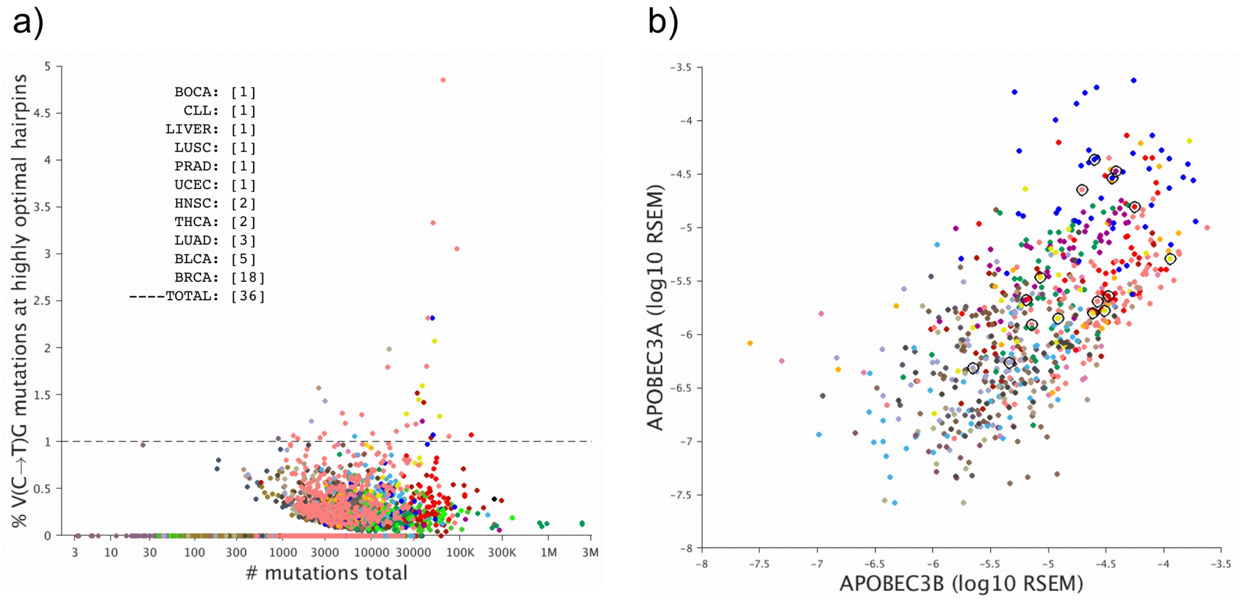

Supp. Fig. 3. Quantification of the extended APOBEC signature (VpC hairpin signature) at CpG dinucleotides. (a) For each WGS patient, we examined C->T mutations in VCG trinucleotides and calculated the percent that are in highly optimal hairpins (stem strength ≥ 12 in 3/3 loops, or stem strength ≥ 14 in 4/4 or 4/5 loops). We plotted this metric against the total number of mutations in each sample. A small number of samples (36 out of 2800 total) exceeded a 1% threshold. These were half breast samples, followed by bladder and lung cancers. These are all tumor types that tend to have high levels of APOBEC expression. (**b)** Per-patient expression levels of A3A and A3B are highly correlated. Circles indicate patients from the ≥1% set in panel A for which TCGA expression data was available. Some, but not all, of these patients have high APOBEC expression, consistent with our previous report[1] that APOBEC expression levels correlate poorly with APOBEC mutation signature levels. Source data are provided as a Source Data file.

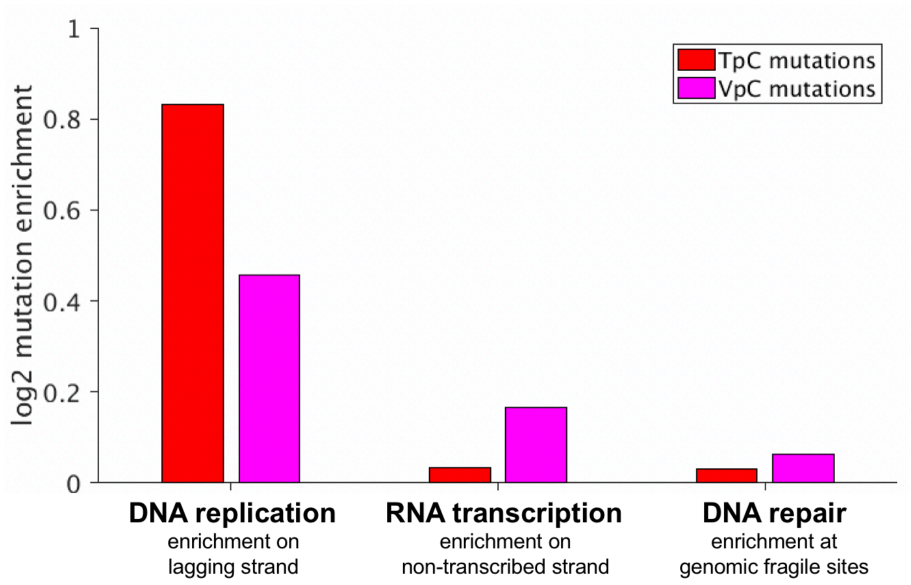

Supp. Fig. 4. Both TpC and VpC mutations show replication-associated strand asymmetry. We applied our published methodology for quantifying mutational strand asymmetry of the replicative and transcriptional types.[2] We analyzed TpC and VpC mutations from the APOBEC+ cohort, applying the *frac_apobec* ≥ 90% cutoff to include only the purest-APOBEC tumors. TpC mutations showed strong replicative strand asymmetry, with ~1.7 as many mutations occurring on the LGST compared to the LDST, as reported previously by us and others[2-5]. In comparison, TpC mutations showed weak transcriptional asymmetry, with less than a 5% increase of mutations on the nontranscribed strand, compared to the transcribed strand. VpC mutations showed a similar pattern: strong replicative asymmetry and weak transcriptional asymmetry. We also considered the possible role of DNA repair, measuring the enrichment of TpC and VpC mutations in fragile regions of the genome[6], but found no difference from non-fragile regions. Source data are provided as a Source Data file.

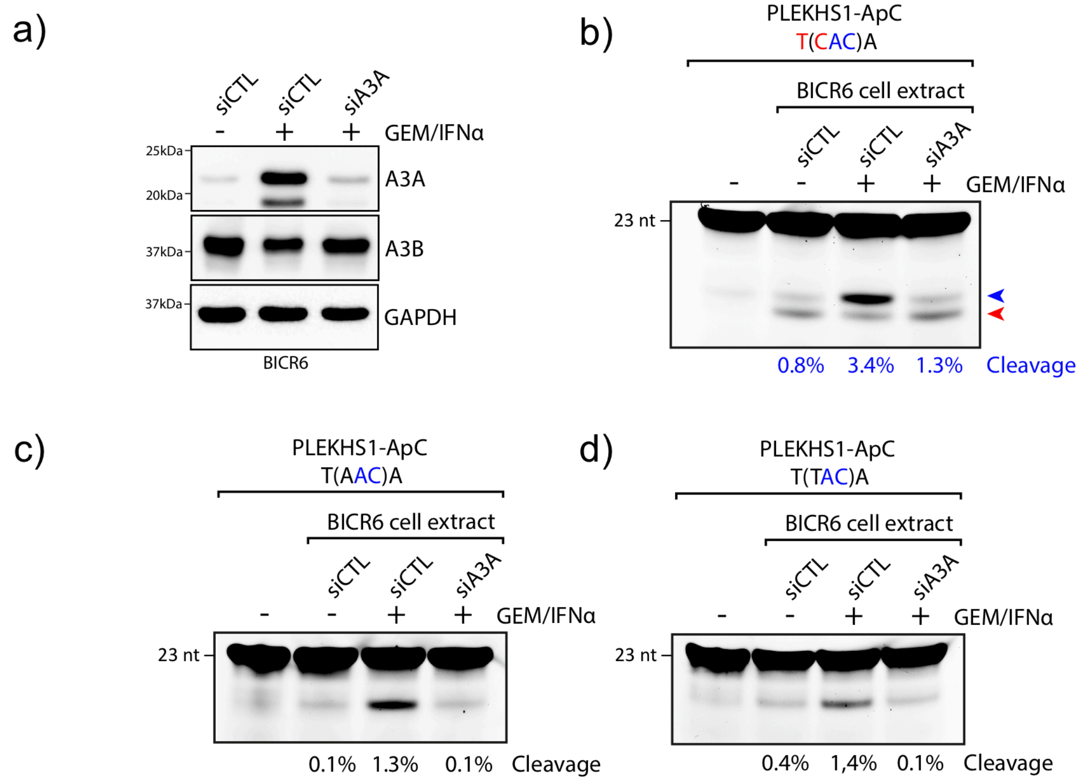

Supp. Fig. 5. Additional experimental evidence supporting APOBEC3A (A3A) as the source of cytosine deamination activity at VpC sites in hairpin loops. (a) Endogenous APOBEC expression in the human pharyngeal squamous cell carcinoma cell line BICR6. At baseline, BICR6 cells express high levels of A3B but low levels of A3A (left lane). However, stimulating the cells with a combination of gemcitabine and interferon alpha (GEM/IFNα) induces strong expression of endogenous A3A (middle lane). Upon co-administration of a small interfering RNA against A3A (siA3A), detected levels of A3A return to near baseline levels (right lane). GAPDH is included as a loading control. (**b)** Activity at VpC hairpin site is due to A3A, not A3B. BICR6 cell extracts were incubated with PLEKHS1-ApC-T(CAC)A, the same substrate as in the rightmost lane of Fig. 2C. Without GEM/IFNα stimulation, only 0.8% of the substrate was cleaved (lane #2), and this was mostly at the TpC site in the substrate (red arrow). GEM/IFNα treatment, which induces A3A, caused a dramatic increase in cleavage, overwhelmingly at the ApC site located at the optimal hairpin loop site (blue arrow). Co-treating with siA3A reduced cleavage to near baseline, showing that A3A is responsible for cleavage at the hairpin ApC site. **(c) and (d)** Activity at VpC hairpin sites in substrates lacking any TpC site. Versions of the PLEKHS1-ApC-T(CAC)A substrate were tested that have an A (panel c) or a T (panel d) at the first position of the hairpin loop. In each case, a strong increase in substrate cleavage was seen upon stimulation of cells with GEM/IFNα, which induces endogenous A3A expression, and the effect was abrogated by co-treatment with siA3A, showing that A3A is responsible for cleavage at these VpC hairpin sites. **(Supp. Fig. 5 continues on next page)**

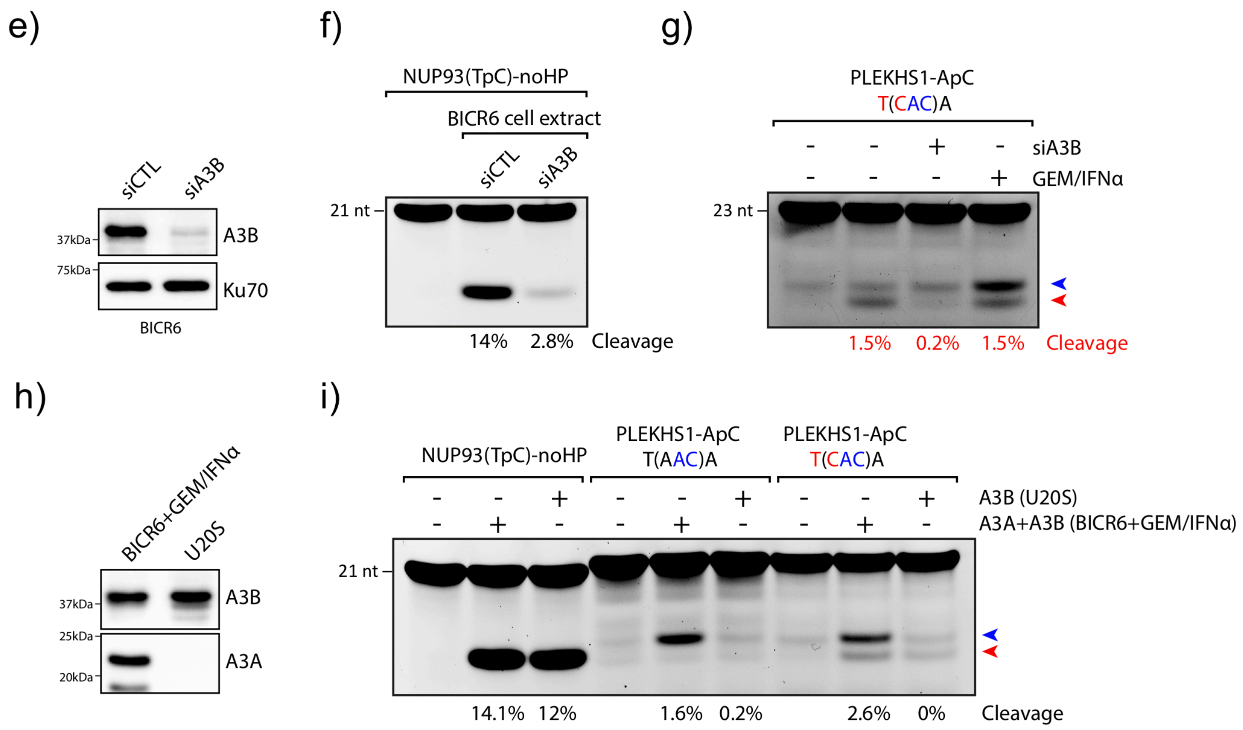

Supp. Fig. 5. (continued) (e) Endogenous expression of A3B in BICR6 cells can be knocked down almost completely by treatment with an siRNA against A3B (siA3B). **(f)** Activity at TpC site in a non-hairpin substrate is due to A3B. BICR6 cell extracts were incubated with NUP93(TpC)-noHP, the same substrate as in the second lane of Fig. 1. This results in 14% of the substrate being cleaved. Treating cells with siA3B causes an 80% reduction of this effect, showing that A3B is responsible for cleavage at this non-hairpin TpC site. **(g)** Control of cleavage site selection in PLEKHS1-ApC-T(CAC)A. At baseline, endogenous expression of A3B leads to 1.5% substrate cleavage at the TpC site (red arrow). Treatment of cells with siA3B abolishes cleavage at the TpC site, reducing total cleavage to 0.2%. As a control, treatment of cells with GEM/IFNα markedly increases cleavage at the ApC site (3% substrate cleavage) but not at the TpC site (1.5% substrate cleavage as for untreated cell extract) **(h)** Comparison of endogenous A3A expression in cell extracts of two cell line models (left lane, BICR6 cells stimulated with GEM/IFNα; right lane, U2OS cells under baseline conditions). BICR6 cell extract (7.5 μg) and U2OS cell extract (15 μg) were normalized to have similar levels of A3B. **(i)** The non-hairpin TpC site in NUP93(TpC)-noHP (left set of lanes) can be efficiently cleaved by cells expressing either A3B alone, or A3A+A3B. However, the hairpin VpC site in the substrate PLEKHS1-ApC-T(AAC)A (middle set of lanes) requires A3A for cleavage. Similarly, the hairpin VpC site in the substrate PLEKHS1-ApC-T(CAC)A (right set of lanes) requires A3A for cleavage. Source data are provided as a Source Data file.

a)

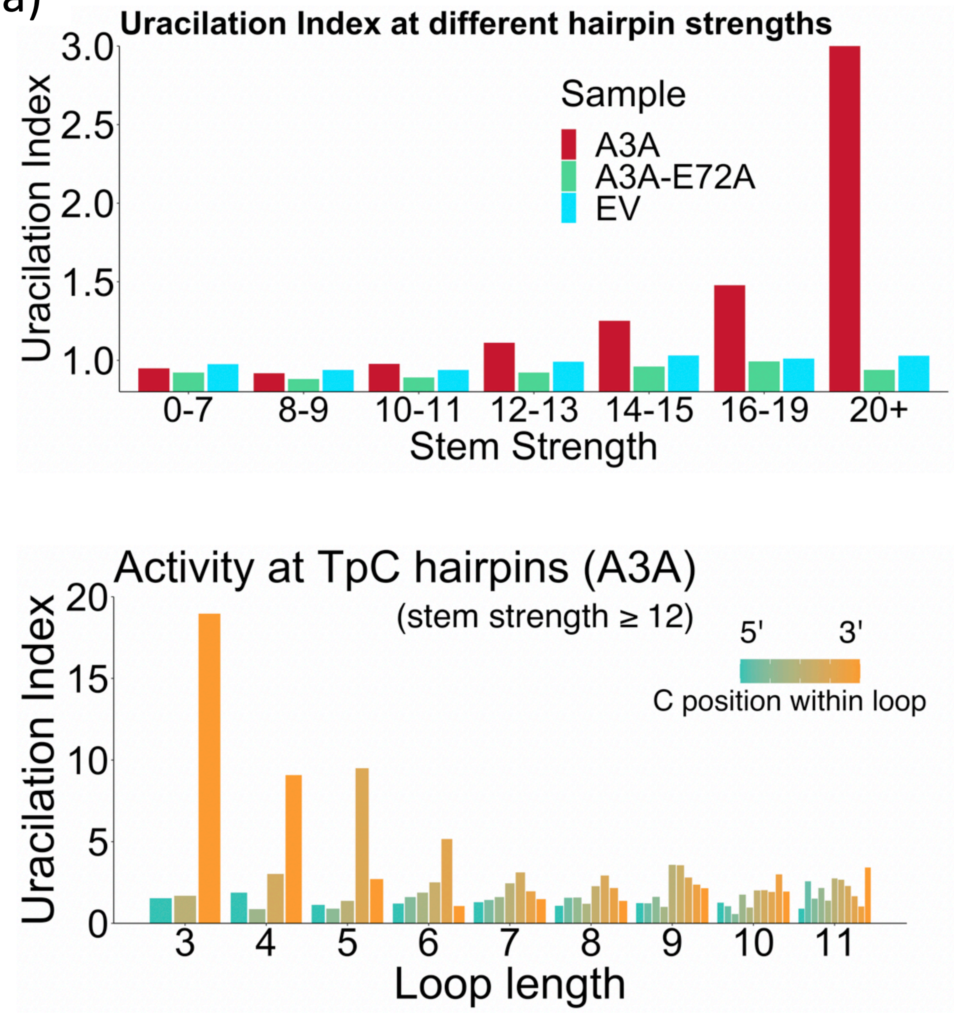

Supp. Fig. 6. APOBEC3A shows deamination activity at TpC sites in *E. coli.* **(a)** APOBEC3A activity at TpC sites (quantified by uracilation index as in Fig. 4) increases with hairpin strength (top panel, red bars), and is abolished by switching to a catalytically inactive A3A point mutant (A3A-E72A, green bars) or an empty expression vector (blue bars). Activity is strongest in 3-nt loops (bottom panel) and decreases in larger loops, and activity is highly position-dependent, as reported previously[7]. (**Supp. Fig. 6 continues on next page.)**

b)

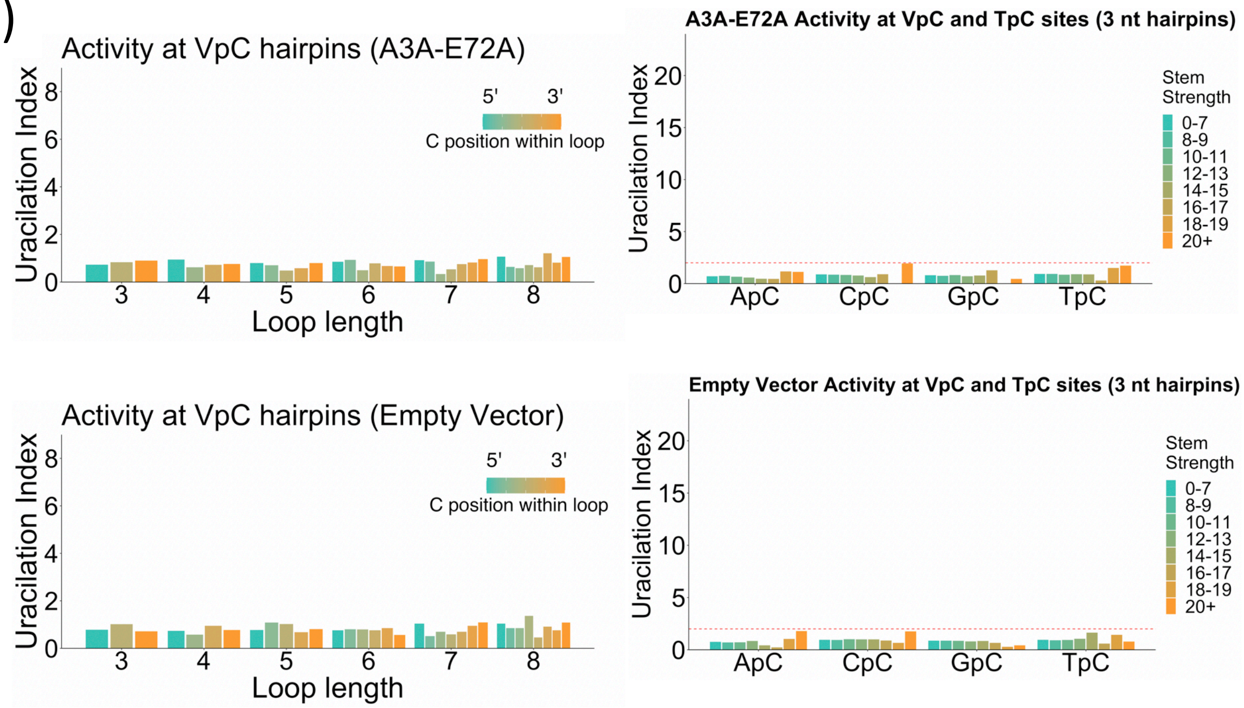

Supp. Fig. 6. (continued) **(b)** Negative control experiments for *E. coli* experiments (LGST and LDST combined). Activity is abolished when using a catalytically inactive A3A point mutant (top row) or empty expression vector (bottom row). Source data are provided as a Source Data file.

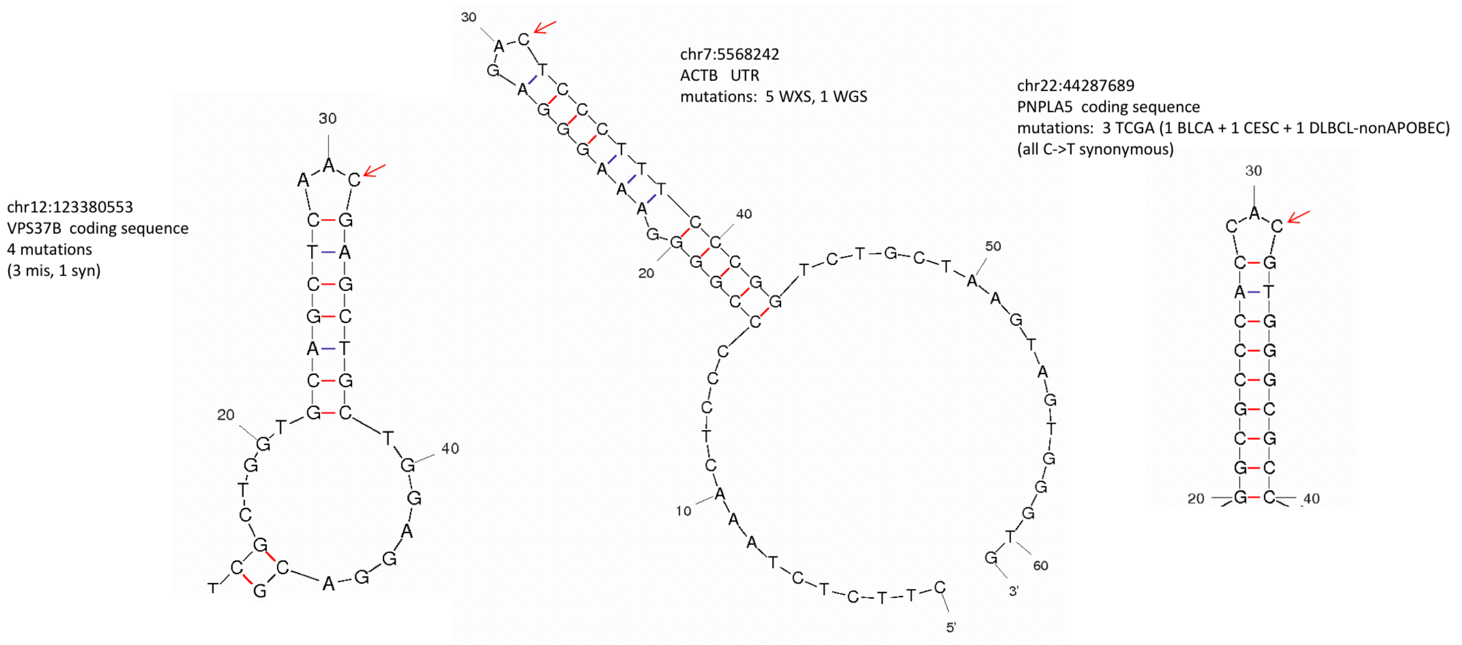

Supp. Fig. 7. Recurrently mutated VpC hairpin sites in APOBEC+ tumors. Three examples are shown of VpC sites in optimal hairpins that are mutated in multiple patients in the WGS cohort.

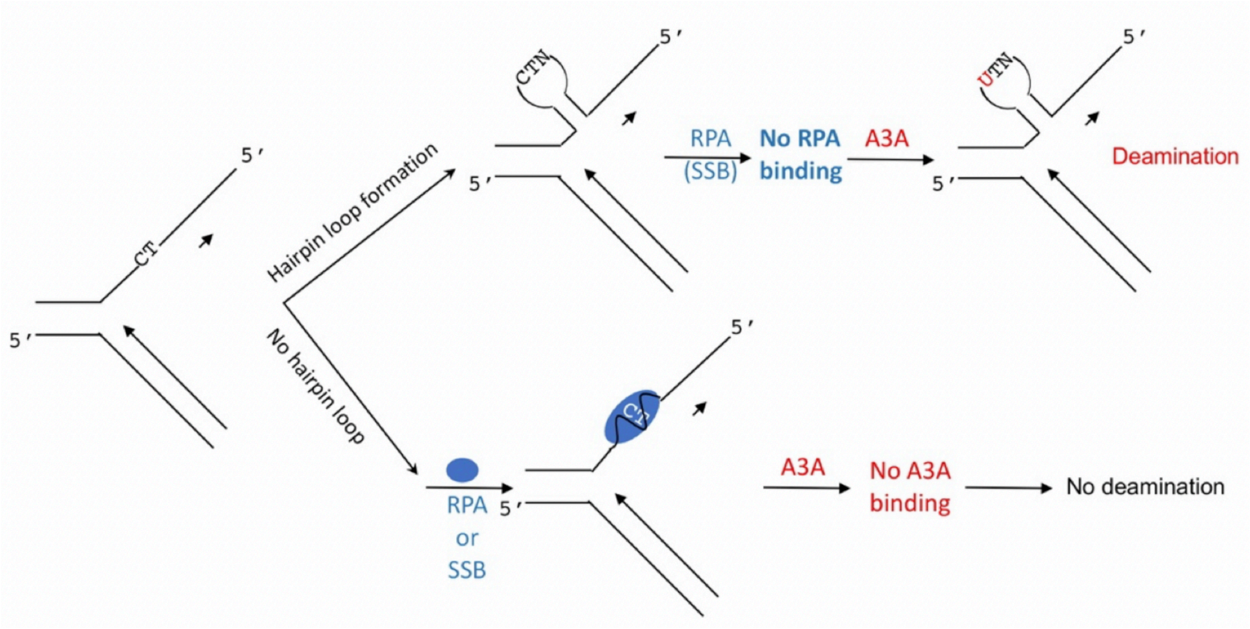

Supp. Fig. 8. Model of the effect of strong hairpin loop formation on RPA binding and cytosine deamination. A stable hairpin in the lagging-strand template (LGST) prevents binding by RPA and allows A3A to deaminate cytosines. In the absence of a stable hairpin, RPA binds ssDNA in the LGST and prevents A3A from deaminating the cytosine.

Supplementary references

1 Jalili, P. *et al.* Quantification of ongoing APOBEC3A activity in tumor cells by monitoring RNA editing at hotspots. *Nature communications* **11**, 2971, doi:10.1038/s41467-020-16802-8 (2020).

2 Haradhvala, N. J. *et al.* Mutational Strand Asymmetries in Cancer Genomes Reveal Mechanisms of DNA Damage and Repair. *Cell* **164**, 538-549, doi:10.1016/j.cell.2015.12.050 (2016).

3 Bhagwat, A. S. *et al.* Strand-biased cytosine deamination at the replication fork causes cytosine to thymine mutations in Escherichia coli. *Proc Natl Acad Sci U S A* **113**, 2176-2181, doi:10.1073/pnas.1522325113 (2016).

4 Hoopes, J. I. *et al.* APOBEC3A and APOBEC3B Preferentially Deaminate the Lagging Strand Template during DNA Replication. *Cell Rep* **14**, 1273-1282, doi:10.1016/j.celrep.2016.01.021 (2016).

5 Seplyarskiy, V. B. *et al.* APOBEC-induced mutations in human cancers are strongly enriched on the lagging DNA strand during replication. *Genome Res* **26**, 174-182, doi:10.1101/gr.197046.115 (2016).

6 Kumar, R. *et al.* HumCFS: a database of fragile sites in human chromosomes. *BMC Genomics* **19**, 985, doi:10.1186/s12864-018-5330-5 (2019).

7 Buisson, R. *et al.* Passenger hotspot mutations in cancer driven by APOBEC3A and mesoscale genomic features. *Science* **364**, doi:10.1126/science.aaw2872 (2019).
